# Supplementary material for: Benchmarking DNA isolation kits used in analyses of the urinary microbiome
Source: Sci Rep. 2021 Mar 17;11:6186. doi: 10.1038/s41598-021-85482-1 (PMC7969918; doi:10.1038/s41598-021-85482-1)
Supplement: Supplementary file 1 — Supplementary information. [file 41598_2021_85482_MOESM1_ESM.pdf]

Supplemental Material to '*Benchmarking DNA isolation kits used in analyses of the urinary microbiome'* by Lisa Karstens, Nazema Y. Siddiqui, Tamara Zaza, Alecsander Barstad, Cindy L. Amundsen, Tatyana A. Sysoeva.

Table S1. Diverse DNA isolation methods utilized in analyses of microbial composition of lower urinary tract.

Study	Urine Collection Method	Volume	DNA Extraction Kit	Lysis Method	DNA Collection Method
Nelson 2010	Void	<50mL	DNeasy Blood and Tissue Kit	Enzymatic	Silica spin column
Dong 2011	Urethral swab or void	1mL or 5mL	DNeasy Tissue Extraction Kit	Enzymatic	Silica spin column
Siddiqui 2011	Void	30mL	DNeasy Blood and Tissue Kit	Enzymatic	Silica spin column
Fouts 2012	Void or catheterization	--	Custom with lysozyme/bead lysis and phenol extraction	Enzymatic and bead beating	Ethanol precipitation
Wolfe 2012	Void, aspiration, and catheterization	--	DNeasy Tissue Extraction Kit	Enzymatic	Silica spin column
Lewis 2013	Void	2mL	Custom with SDS/bead lysis and alcohol precipitation	Bead beating	Isopropanol and ethanol precipitation
Hilt 2014	Catheterization	1mL	DNeasy Blood and Tissue Kit	Enzymatic	Silica spin column
Pearce 2015	Catheterization	1mL	HMP (Yuan 2012) and DNeasy Blood and Tissue Kit	Enzymatic	Silica spin column
Shoskes 2016	Void	--	PowerMag Microbiome RNA/DNA Isolation Kit	Bead beating	Magnetic beads
Karstens 2016	Catheterization	50mL	DNeasy Blood and Tissue Kit	Enzymatic	Silica spin column
Modena 2017	Void	50mL	Custom with TRIzol	TRIzol Reagent	Alcohol precipitation
Thomas-White 2017	Void or catheterization	1mL	DNeasy Blood and Tissue Kit	Enzymatic	Silica spin column

Gottschick 2017	Void	15mL	Phenol extraction with consecutive peqGOLD Tissue DNA Kit	Bead beating	Silica spin column
Liu 2017	Modified void	40mL	Custom with PowerMag Microbiome RNA/DNA Isolation Kit	Bead beating	Magnetic beads
Popovic 2017	Void	30mL	PowerSoil DNA Isolation Kit	Bead beating	Silica spin column
Wu 2017	Catheterization	50mL	DNeasy Blood and Tissue Kit	Enzymatic	Silica spin column
Burton 2017 (dog urine)	Aspiration (cystocentesis)	30mL	Custom with QIAamp	Bead beating	Silica spin column
Adebayo 2017	Void	1mL	DNeasy Blood and Tissue Kit	Enzymatic	Silica spin column
Shrestha 2018	--	30mL	Custom with phenol extraction	Enzymatic and bead beating	Alcohol precipitation
Jung 2019	Void	200µL	DNeasy Powersoil Kit	Bead beating	Silica spin column
Pohl 2020	Void and catheterization	--	DNeasy or QIAmp DNA Micro Kit	Enzymatic	Silica spin column
Forster 2020	Catheterization	>5mL	QIAamp Circulating Nucleic Acid Kit with QIAamp columns	Enzymatic	Silica column
Kinneman 2020	Catheterization	>1mL	EZ1 DSP Virus Kit	Enzymatic	Silica magnetic beads

Table S2. Approximate volumes* of voided urine used in study.

Sample	Volume
1	30 mL
2	30 mL
3	50 mL
4	75 mL
5	75 mL
6	50 mL
7	75 mL
8	75 mL
9	100 mL
10	100 mL
11	100 mL

**Volume estimates were measured in the cups provided, without additional transfer into measuring cylinders to avoid contamination and therefore, these are estimates that are rounded to closest measuring mark.*

Table S3. Recovered concentrations after DNA isolation

**Sample/kit**	Kit 1 (BiOstic)	Kit 2 (Blood&Tissue)	Kit 3 (Promega)	Kit 4 (PowerSoil)	Kit 5 (UltraClean)
1	0.255	1.107	0.000	0.012	0.032
2	0.282	0.584	0.000	0.126	0.101
3	4.763	9.402	0.578	4.563	1.576
4	0.995	2.386	0.066	0.808	0.695*
5	2.046	4.403	0.063	1.138	0.001
6	0.101	0.395	0.000	0.040	0.037
7	0.804	1.027	0.000	0.137	0.269
8	1.153	5.752	0.121	0.906	0.751
9	0.549	1.333	0.078	0.265	0.331
10	1.362	3.073	0.240	0.745	0.596
11	0.007*	0.055	0.000	0.004*	0.000*
PBS control	0.000*	0.008	0.000	0.005*	0.007*

DNA concentrations in ng/μL

* - *no agarose gel band detected after PCR amplification*

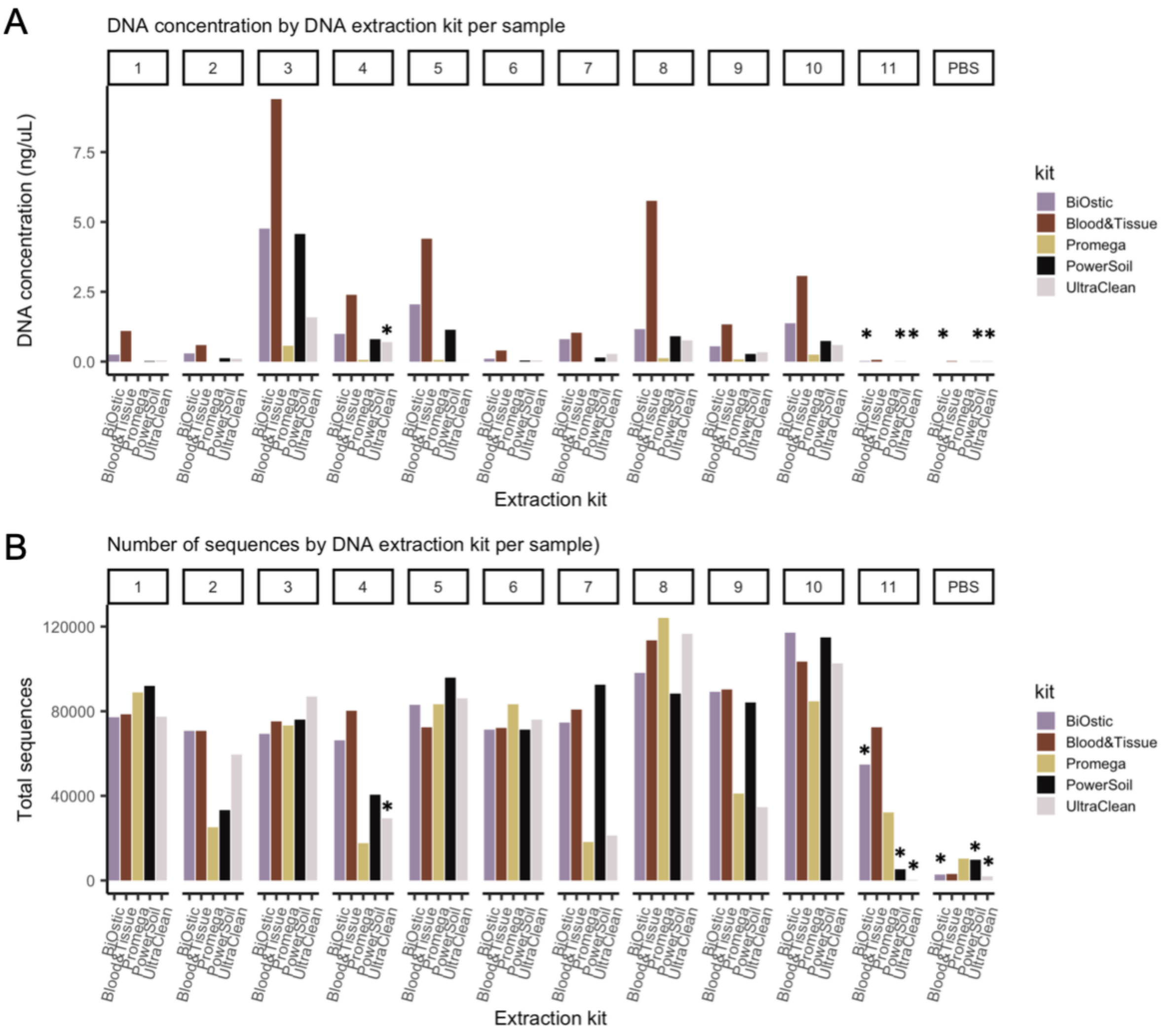

Figure S1. Comparative data for all samples. A. Comparing the amount of isolated DNA from urine. Asterisks indicate samples that did not produce identifiable PCR product. **B.** Comparing the number of sequencing reads obtained from DNA isolated with each kit. Samples 1 - 11 represent DNA extracted from voided urine while Sample 12 is a negative control of filtered sterile PBS solution. Asterisks in both panels mark samples that did not yield detected gel bands after amplification of isolated DNA with primers specific for the V4 region of the 16S rRNA gene.

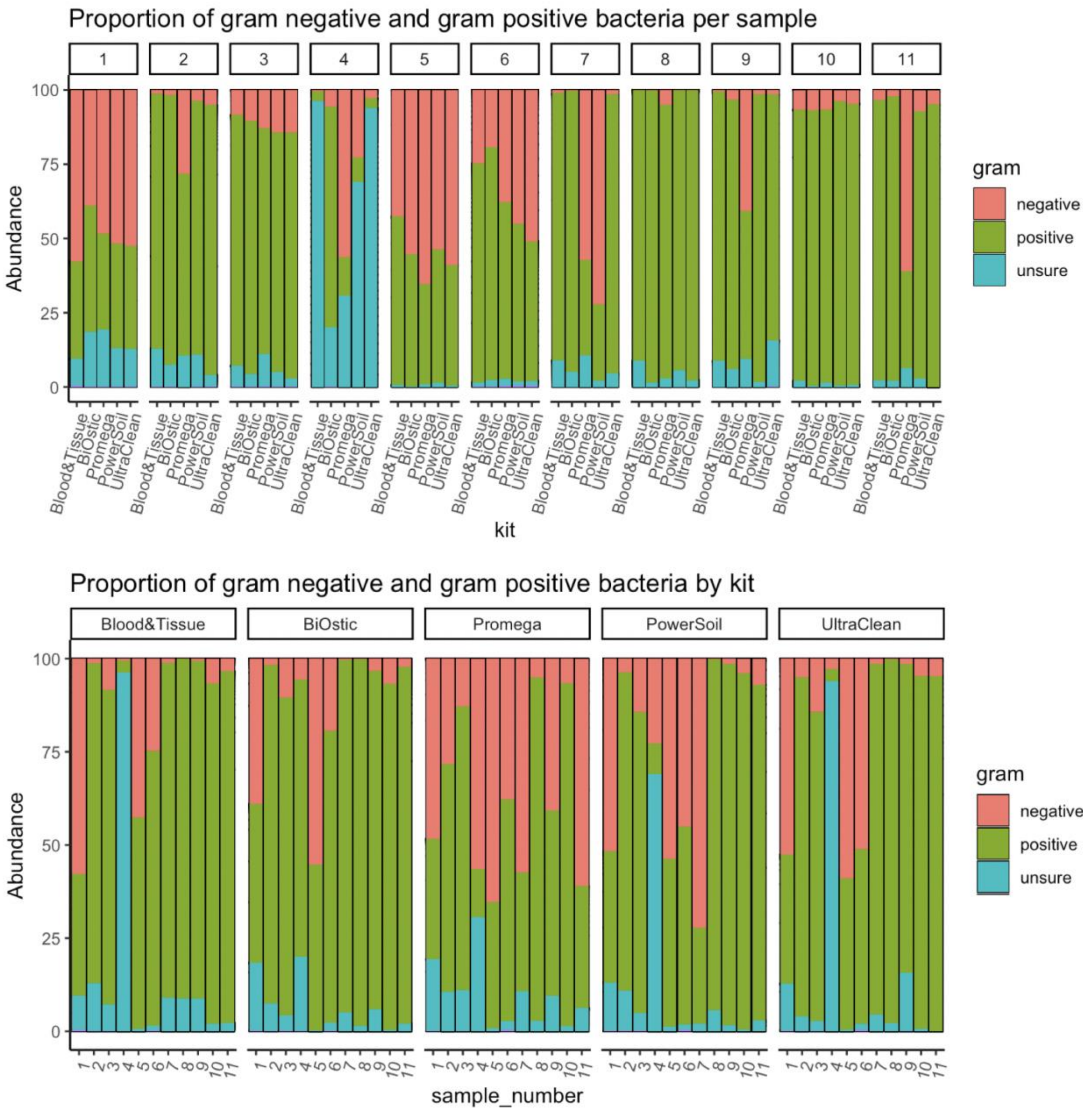

Figure S2. Relative abundance of Gram positive and Gram negative bacteria. Data displayed per sample and also per kit. The summary of these data is presented in Figure 5.

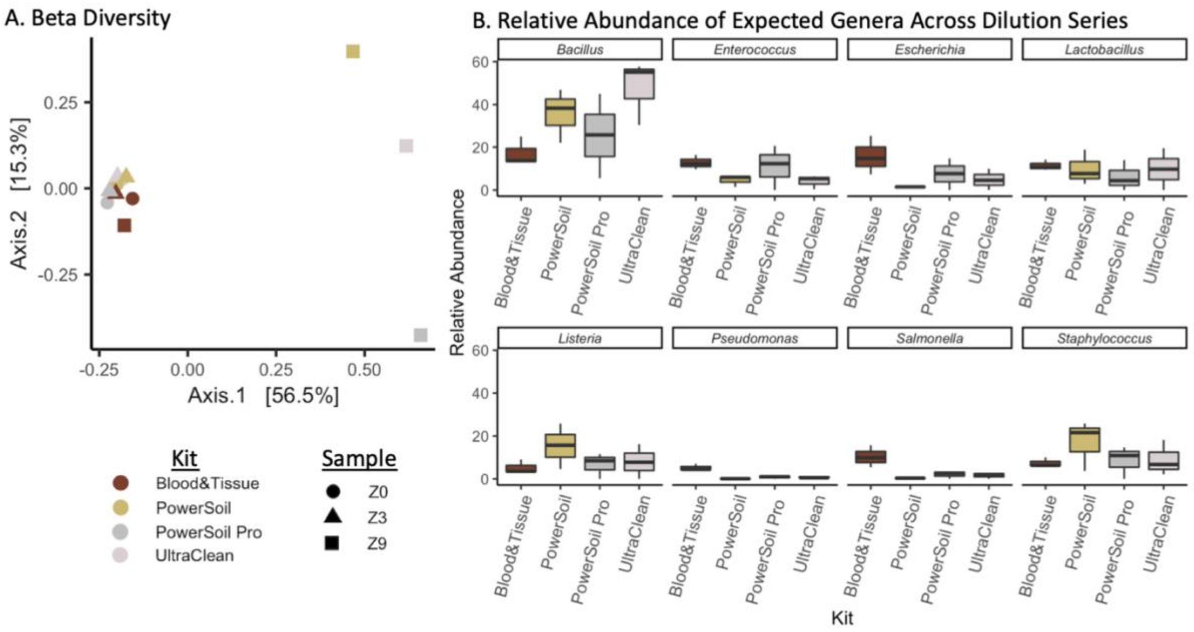

Figure S3. Beta Diversity (A) and relative abundance (B) of expected bacteria from a mock microbial community dilution series. A well characterized mock microbial community (ZymoBIOMICS Microbial Community Standard, Zymo Research, Irvine, CA) was tested using a subset of DNA isolation kits from the primary study. Due to technical and logistic issues in our laboratory, we were unable to re-test the Promega or Qiagen BiOstic kits in this follow up experiment. The Qiagen DNeasy Blood & Tissue, Qiagen DNease PowerSoil, and Qiagen DNeasy UltraClean were utilized according to their respective protocols with the same methods as described in the primary study. Though the PowerSoil kit was tested here, it has recently been replaced by the PowerSoil Pro kit, which was also tested in this follow up experiment. A mock microbial community was prepared as a dilution series by performing 9 rounds of a three-fold dilution with microbe-free water. The undiluted (Z0), third (Z3) and final (Z9) dilutions were subject to DNA extraction, PCR amplification, and 16S rRNA gene sequencing. **A.**The beta diversity analysis demonstrates that overall results from mock community samples are similar despite the DNA isolation kit that is used. However, for the most dilute (Z9) samples, only the Blood & Tissue kit revealed observed sequence data similar to less dilute samples. With other kits, the results are highly variable and distinct from less dilute samples. **B.** Relative abundances of expected genera across all dilutions are depicted. The median and full range of bacterial abundance is shown. When considering all samples (undiluted and diluted) in aggregate, there were no significant differences in recovery of expected bacteria between DNA isolation kits, and no confirmed patterns of bias in Gram negative or Gram positive bacterial recovery.

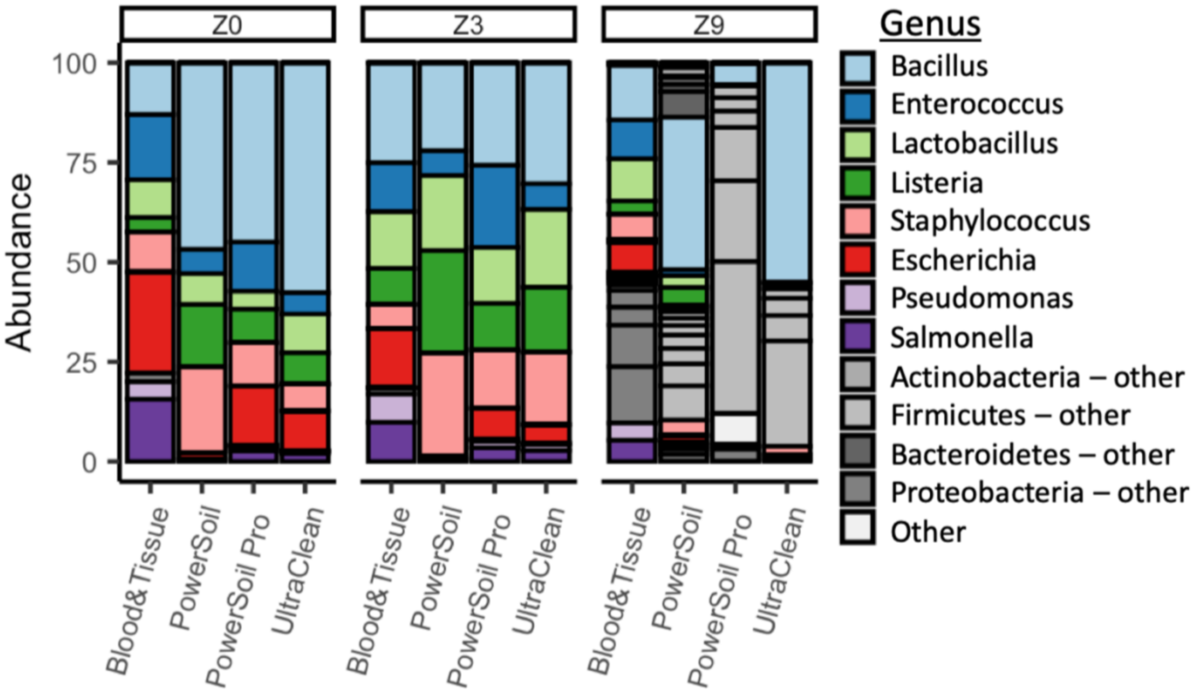

Figure S4. Stacked bar plots showing relative abundance of expected bacteria from the mock microbial community dilution series. A mock microbial community (ZymoBIOMICS Microbial Community Standard, Zymo Research, Irvine, CA) was prepared as a dilution series by performing 9 rounds of a three-fold dilution with microbe-free water. The 8 expected bacteria from this community are depicted in colors, and other bacteria (presumed contaminants) are depicted in shades of gray. Undiluted (Z0) and slightly diluted (Z3) samples revealed expected results after sequencing. However, for the most dilute sample (Z9), all 8 expected bacteria were only observed when DNA was isolated with the Qiagen DNeasy Blood & Tissue kit. Sequencing results from all kits showed contaminants that were not seen with less dilute samples. However, with the PowerSoil, PowerSoil Pro and UltraClean kits, only certain bacteria were recovered, with a much larger proportion of contaminants.
